# An Atypical Presentation of Morel–Lavallée Syndrome Following Blunt Trauma

**DOI:** 10.7759/cureus.101714

**Published:** 2026-01-16

**Authors:** Shreya Nair, Anoop V Pillai, Riju Ramachandran

**Affiliations:** 1 General Surgery, Amrita Institute of Medical Sciences and Hospital, Kochi, IND

**Keywords:** blunt trauma, closed degloving injury, early drainage, medial thigh, morel-lavallée syndrome, post-traumatic swelling, ultrasonography

## Abstract

Morel-Lavallée syndrome (MLS) refers to a closed internal degloving injury resulting from tangential shearing forces, which cause separation of the skin and subcutaneous layers from the underlying deep fascia, with subsequent collection of hemolymphatic fluid and devitalized adipose tissue in the created potential space. Despite its rarity, MLS contributes substantially to soft-tissue shear injuries and is commonly underdiagnosed, with consequent delays in treatment and related complications.

A 44-year-old female reported localized pain with a progressively enlarging swelling involving the inferomedial aspect of the right thigh subsequent to a road-traffic accident. Clinical examination revealed a large, well-defined, fluctuant, non-tender swelling with overlying bluish-black discoloration and no neurovascular compromise. Ultrasonography reported a well-circumscribed fluid collection located between the subcutaneous adipose tissue and the underlying fascia, with no associated bony injury on radiography. Given a high index of suspicion for MLS, early surgical intervention was undertaken within 72 hours of injury. Incision and drainage revealed clear separation of the subcutaneous tissue from the fascia, with evacuation of approximately 200 cc of hematoma. The postoperative course was uneventful, and secondary suturing was performed on day five, with no evidence of infection or residual collection during the inpatient stay.

MLS commonly involves the peri-trochanteric region but may present at atypical sites such as the medial thigh. The lesion can mimic cellulitis, abscess, or hematoma, underscoring the importance of clinical suspicion. Previous literature indicates that ultrasonography is useful for the early detection of Morel-Lavallée lesions, whereas magnetic resonance imaging is preferred for the evaluation of established or chronic lesions. Evidence derived mainly from retrospective series and narrative reviews suggests that early percutaneous or surgical drainage has been associated with lower rates of recurrence and morbidity; however, outcomes may vary, and the quality of evidence remains limited. In patients with persistent or recurrent collections, definitive surgical management such as capsulectomy, minimally invasive approaches, or sclerotherapy, with doxycycline emerging as an effective sclerosant, has been described.

This case report highlights the importance of maintaining a high index of suspicion for MLS in atypical clinical presentations and demonstrates that early recognition, followed by timely drainage and structured postoperative care, may help prevent progression to chronicity, recurrence, and secondary infection.

## Introduction

Morel-Lavallée syndrome (MLS), originally described by Victor-Auguste-François Morel-Lavallée in 1863, is a closed post-traumatic internal degloving injury resulting from tangential shearing forces that detach the skin and subcutaneous tissues from the underlying deep fascia, leading to the formation of a potential space that progressively fills with lymph, necrotic fat, and blood. Although uncommon, MLS represents a clinically relevant subset of soft-tissue shear injuries described in the trauma literature, with the true incidence likely under-recognized because it is frequently misdiagnosed as cellulitis, abscess, contusion, or simple hematoma [1,2].

The pathophysiology follows a characteristic sequence. The shearing mechanism causes disruption of perforating vessels and lymphatic channels, resulting in the accumulation of blood and serosanguinous fluid within the newly created cavity. Macroscopic evaluation commonly demonstrates blood clots, fibrinous material, and a mixture of viable and necrotic adipose tissue, and bacterial colonization has been documented in up to 46% of cases, irrespective of the interval between injury and surgical debridement [3]. Lesion evolution progresses through four stages: (1) separation of dermis from fascia, (2) acute fluid collection, (3) progressive enlargement with serosanguinous transformation, and (4) chronicity with pseudo-capsule formation [4].

The anatomical distribution of MLS reflects regions with increased skin mobility, large surface area, and rich vascular networks. In a review of more than 200 cases, Vanhegan et al. reported that lesions most commonly involved the greater trochanteric/hip region, followed by the thigh, pelvis, knee, gluteal region, and lumbosacral area, with less frequent involvement of the calf, abdomen, and head; the peritrochanteric region was the predominant site of occurrence [5].

MLS is clinically significant because of its association with perioperative complications, particularly in pelvic and acetabular trauma. Suzuki et al. demonstrated that MLS acts as an independent predictor of postoperative surgical-site infection, whereas Kim et al. found no statistically significant association between MLS and an increased risk of infection [6,7]. Currently, no universally accepted treatment algorithm exists, emphasizing the importance of early diagnosis and individualized management [2,8]. This case report was presented as an oral presentation at the Spasht Undergraduate Medical Conference on August 30, 2025.

MLS is frequently misdiagnosed as cellulitis, abscess, or simple hematoma, resulting in diagnostic delay and inappropriate management. This case highlights critical decision points-early suspicion, timely imaging, and prompt intervention-that influence chronicity, infection risk, and recurrence. The reported rates of misdiagnosis and bacterial colonization reflect heterogeneous trauma populations and indicate the biologically active nature of MLS lesions rather than inevitable infection, underscoring the importance of early recognition before chronic encapsulation develops.

## Case presentation

A 44-year-old woman presented to the emergency department around midnight with localized pain and swelling over the inferomedial region of the right thigh following a road-traffic accident earlier that evening. She was riding a scooter when she was struck by an auto-rickshaw, causing her to fall on her right side, with the scooter landing directly on her lower limb.

Subsequently, she noticed the development of a swelling in the lower inner thigh, initially small but gradually increasing in size over the next few hours. She reported a throbbing type of pain, exacerbated by walking and relieved by rest, resulting in difficulty ambulating.

There were no associated symptoms such as fever, sensory deficits in the lower limb, or any external discharge.

**Figure 1:**
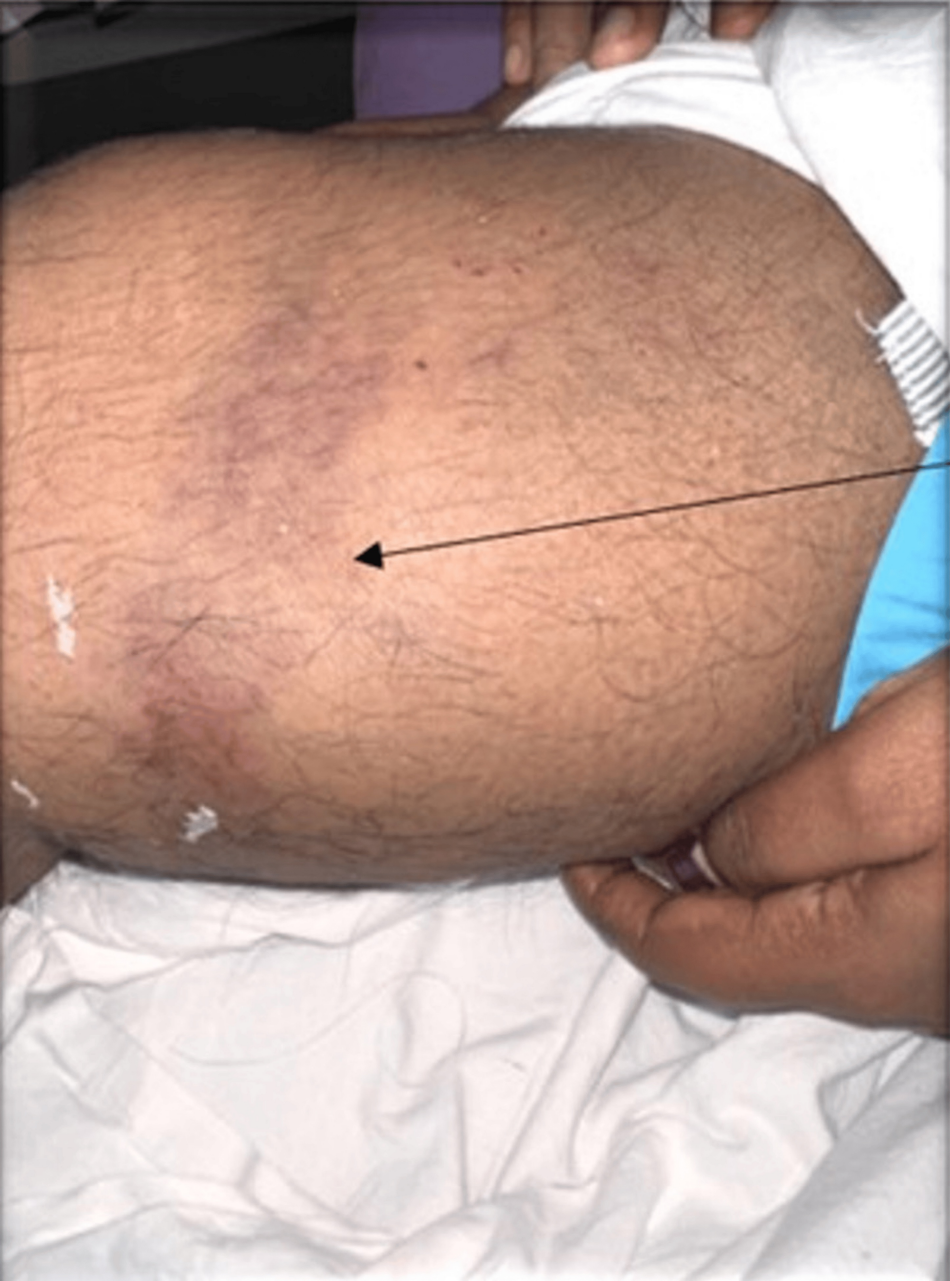
The clinical picture at presentation Bluish-black area over the lower thigh (marked with arrow).

On examination of the right thigh revealed a large, well-defined, oval swelling over the lower medial aspect of the thigh, measuring approximately 15 × 15 cm on inspection and 15 × 20 cm on palpation. The overlying skin showed bluish-black discoloration, with a smooth surface and clearly demarcated margins. On palpation, there was no local rise of temperature; the lesion was soft, fluctuant, and non-tender, without adherence to the overlying skin, and its size did not change with muscle contraction, indicating an extra-muscular collection. There were no visible pulsations, dilated veins, or audible bruits, suggesting a non-vascular swelling (Figure 1). There was no restriction in joint mobility. She was reviewed in the morning rounds, and an ultrasound of the thigh was ordered.

**Figure 2:**
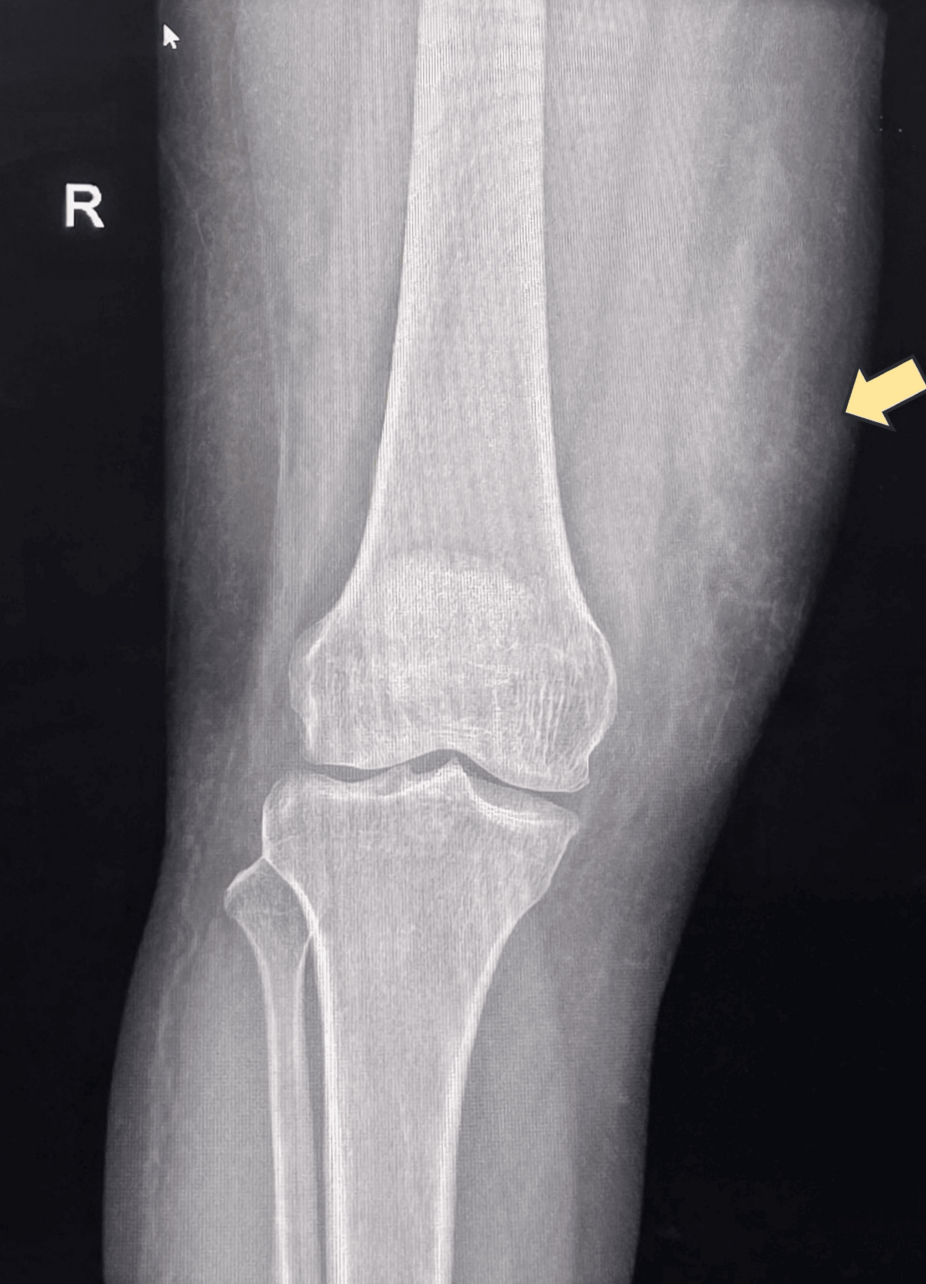
X-ray of the lower thigh, knee, and leg The arrow pointing to the Morel-Lavallée lesion.

Ultrasound of the right thigh reported a well-defined, linear fluid collection with internal mobile echoes located between the subcutaneous fat and the underlying fascia. The collection reported to measure approximately 6.7 cm in length and 1.2 cm in maximal thickness. X-ray showed no evidence of underlying bony injury or fracture (Figure 2). Routine laboratory investigations, including inflammatory markers, muscle enzyme levels, and urine analysis for myoglobinuria, were performed and were within normal limits.

**Figure 3:**
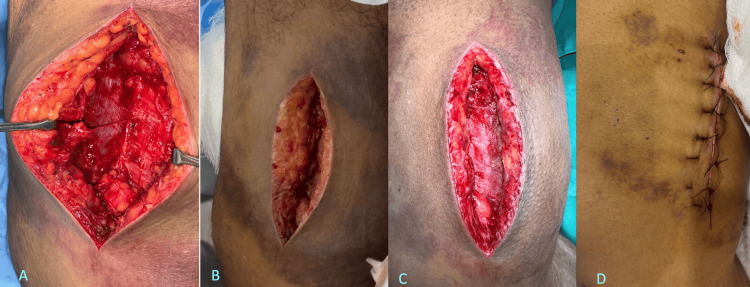
Intraoperative and postoperative wound progression 3a) Intraoperative picture showing the cavity and sheared tissue, 3b) First post-op day, 3c) Third post-op day, 3d) Sutured wound 5th post-op day

The patient underwent an incision and drainage procedure under regional nerve block later that evening. An incision was placed in the lower medial thigh extending deep up to the level of the facial plane without opening the fascia. Intraoperatively, there was clear separation of the subcutaneous fat from the underlying fascial plane, creating a potential space from which approximately 200 cc of hematoma was evacuated. Postoperatively, the wound was assessed on day 3, and showed no signs of infection or residual collection. A secondary suturing was performed on day 5 (Figure 3).

**Figure 4:**
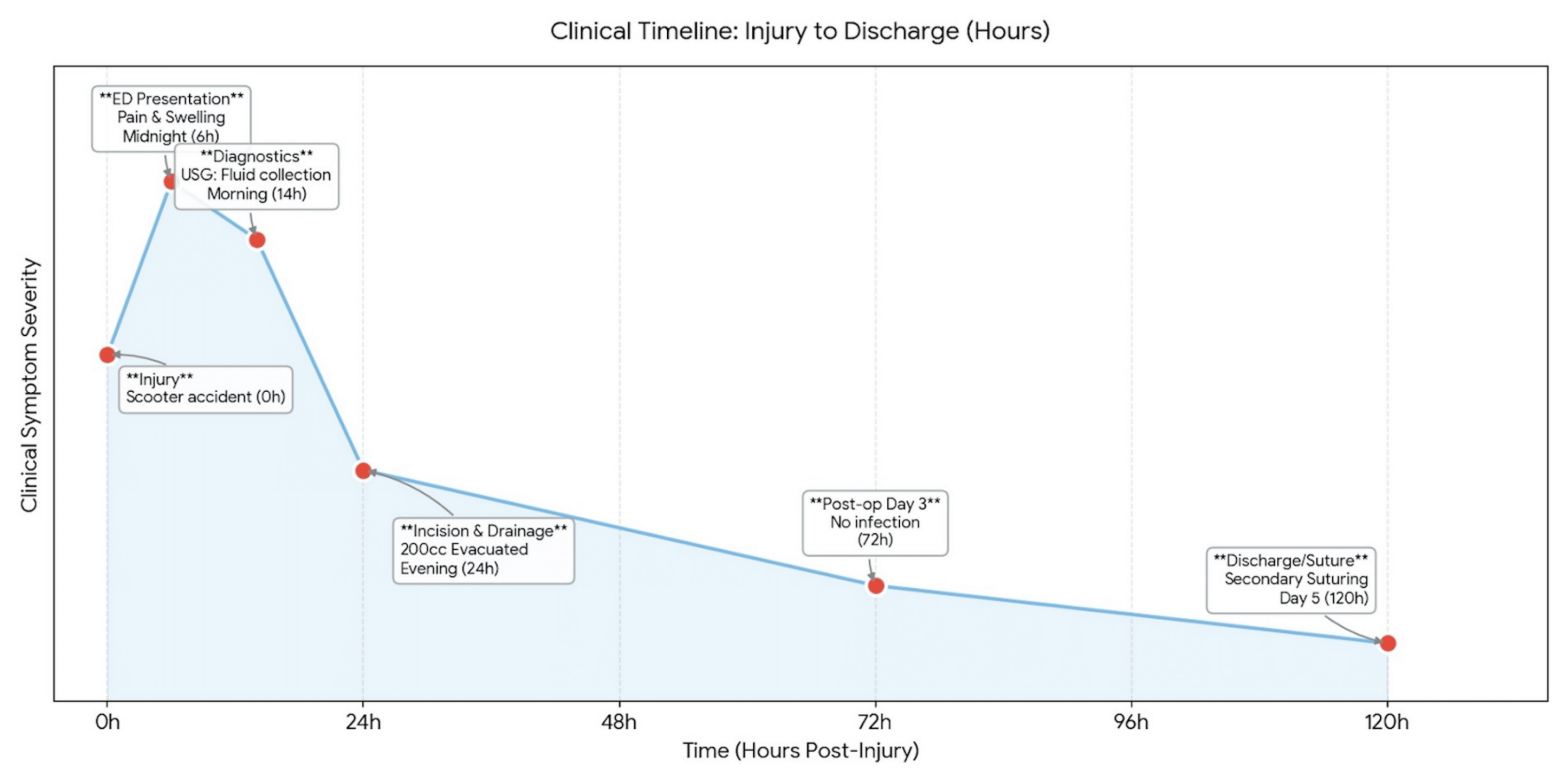
Simplified clinical timeline from injury to discharge Figure illustrating the sequence of key events with a simplified timeline highlighting the progression from initial trauma and symptom onset to diagnosis, surgical intervention, and postoperative recovery.

Given the high index of suspicion for MLS, the patient underwent early incision and drainage under regional nerve block. Intraoperatively, clear separation of subcutaneous fat from the fascial plane was noted, with evacuation of approximately 200 cc of hematoma. Postoperative assessment on day three showed no evidence of infection or residual collection, and secondary suturing was performed on day five. Early recognition and intervention within 72 hours of injury successfully prevented complications associated with chronic Morel-Lavallée lesions (MLLs) (Figure 4).

## Discussion

MLS is an uncommon closed degloving injury produced by tangential shearing forces that separate subcutaneous tissue from the underlying deep fascia, resulting in a potential space filled with hemolymphatic fluid and necrotic fat. Previously reported cases most commonly follow high-energy trauma such as road-traffic accidents or crush injuries, with a predominance in young adult males and frequent association with pelvic, acetabular, or proximal femoral fractures, a combination known to increase postoperative infection risk when unrecognized [1,2]. The greater trochanter and proximal thigh are the most frequently reported anatomical sites, although lesions have also been described around the pelvis, knee, gluteal region, lumbar area, and rarely the upper limb [2,5]. Diagnosis is often delayed, as many lesions present weeks to months after injury as a persistent, fluctuant swelling; chronic lesions commonly develop a fibrous capsule that limits spontaneous resolution. While ultrasound and CT are useful for initial detection, MRI is consistently reported as the imaging modality of choice for lesion characterization, chronicity assessment, and treatment planning, with several authors proposing MRI-based classification systems to guide management [1,2]. Reported treatment approaches range from compression and observation for small acute collections, to percutaneous drainage with or without sclerotherapy for subacute lesions, and open debridement with capsulectomy for chronic, recurrent, or infected lesions [1,2,4,5,8]. Across case series, simple aspiration alone is associated with high recurrence rates, whereas definitive surgical management yields more durable outcomes in chronic MLS.

MLLs are classically reported over the trochanteric and lumbosacral regions; atypical sites, including the medial thigh and scalp, have been documented [2,5]. Because MLLs can clinically mimic cellulitis, abscesses, contusions, hematomas, or soft-tissue tumors, a high index of suspicion is essential, particularly following high-energy trauma [1,4]. Untreated lesions may progress to chronic pseudo-capsule formation, infection, persistent deformity, or skin necrosis [8].

Ultrasound is useful for early detection, while MRI remains the gold standard for defining lesion extent and chronicity [3,9]. Early percutaneous drainage combined with debridement, irrigation, and suction drainage has shown high success rates with low complication profiles when performed within 72 hours of injury [10]. However, aspiration alone carries a high recurrence rate, particularly when volumes exceed 50 mL, warranting operative intervention [8].

Chronic or recurrent lesions often require definitive management. Open capsulectomy with cutaneofascial retention sutures has demonstrated success rates up to 95%, albeit with increased scarring [5]. Minimally invasive approaches, including endoscopic debridement with pseudo capsulectomy and adjuncts such as fibrin glue, offer favorable outcomes with reduced morbidity [8]. Sclerotherapy is increasingly used to obliterate persistent dead space; doxycycline remains the most studied agent with an efficacy of approximately 95.7%, while alternatives such as povidone-iodine may be useful in resource-limited settings [1,2].

Overall, management should be individualized based on lesion chronicity, location, volume, and recurrence risk. Timely diagnosis and appropriate intervention are critical to preventing recurrence and long-term complications [2,3,10].

## Conclusions

MLS is an uncommon yet clinically important closed degloving injury that is frequently overlooked, particularly when occurring at atypical sites such as the medial thigh. This case highlights the importance of maintaining a high index of suspicion in patients presenting with post-traumatic, fluctuant soft-tissue swellings without skin breach. Early ultrasonographic evaluation facilitated prompt diagnosis, and timely surgical intervention in the acute phase led to satisfactory short-term resolution in this patient. While prior studies suggest reduced recurrence with early, stage-appropriate management, longer follow-up is required to determine sustained prevention of chronicity and recurrence. Increased clinician awareness and early recognition remain central to optimizing outcomes and minimizing morbidity.
